# Differences in Biologic Clinical Trials for Chronic Rhinosinusitis With Nasal Polyps—Are We Comparing Apples With Oranges?

**DOI:** 10.1002/alr.70205

**Published:** 2026-07-03

**Authors:** Marjolein Cornet, Peter W. Hellings, Martin Desrosiers, Martin Wagenmann, Richard Follows, Laura Walrave, Luz Adriana Jimenez, Lee Tombs, Dawn Edwards, Peter Howarth, Joseph K. Han

**Affiliations:** ^1^ Department of Otorhinolaryngology Alrijne Hospital Leiderdorp Netherlands; ^2^ Department of Otorhinolaryngology Catholic University of Leuven, and Upper Airways Research Laboratory University of Ghent Ghent Belgium; ^3^ University of Montreal Hospital Center and Research Center Surgery Université De Montréal Montreal Quebec Canada; ^4^ Department of Otorhinolaryngology Düsseldorf University Hospital (UKD) Düsseldorf Germany; ^5^ Clinical Sciences Respiratory, Immunology & Inflammation Research Unit, GSK London UK; ^6^ Global Medical, Specialty Care, GSK Wavre Belgium; ^7^ Global Real‐World Evidence & Health Outcomes Research, GSK London UK; ^8^ Precise Approach Ltd London UK; ^9^ Biostatistics, Respiratory, Immunology & Inflammation Research Unit, GSK London UK; ^10^ Global Medical Affairs, GSK London UK; ^11^ Department of Otolaryngology Head and Neck Surgery Old Dominion University Norfolk Virginia USA

**Keywords:** biomarker, chronic rhinosinusitis, eosinophilic rhinitis and nasal polyposis, therapeutics

## Abstract

In recent years, several biologics targeting Type 2 inflammation have been developed for treating chronic rhinosinusitis with nasal polyps (CRSwNP). These have been studied in registrational randomized controlled trials (RCTs), which vary in their patient populations, trial design, endpoints, geography, timing, or data‐handling processes. While (in)direct treatment comparisons and meta‐analyses have been carried out to compare efficacy results from RCTs, often these fail to properly account for these between‐study differences. Here, we summarize the key between‐study differences that can influence trial outcomes and highlight the resulting challenges faced when comparing outcomes from different Phase III RCTs of biologics in CRSwNP.

## 1 Introduction

Chronic rhinosinusitis with nasal polyps (CRSwNP) is an inflammatory disease characterized by nasal obstruction, loss of smell, and nasal discharge [1]. While the pathophysiology of CRSwNP is complex, it is frequently characterized by Type 2 (T2) inflammation (increased blood/tissue eosinophil counts and immunoglobulin E [IgE] levels) [1, 2], which is associated with high disease burden and risk of recurrence (post‐surgery) [2, 3].

Several biologics have been approved for CRSwNP, including those targeting core cytokines involved in T2 inflammation (e.g., interleukins‐4/‐5/‐13) [4, 5, 6]. However, selecting the right treatment for the patient to obtain the best results can be challenging. The EVEREST Phase IV study [7] was the first head‐to‐head study comparing efficacy between active treatments for CRSwNP and coexisting asthma (dupilumab and omalizumab); however, multiple (in)direct treatment comparisons and meta‐analyses have compared results from Phase III CRSwNP randomized controlled trials (RCTs) [8, 9, 10, 11, 12, 13, 14, 15, 16, 17]. While many aspects of these RCTs are similar and prompt comparisons, other aspects vary substantially, including patient populations, trial design, endpoints, biologic dosing regimens (ranging from every 2 weeks to every 26 weeks), geography, timing, or data‐handling. As these different factors may influence trial outcomes, (in)direct comparisons may not be appropriate as they do not account for these underlying differences [18]. Although statistical approaches exist that can somewhat adjust for confounding factors, such as propensity weighting, a matching‐associated indirect comparison [19, 20], or identification of comparable subgroups between trials [21], if the study populations or designs differ considerably or sufficient patient‐level data are unavailable, these approaches can still lead to inaccurate and/or misleading between‐study comparisons.

The aim of this article is to highlight the challenges faced when comparing (directly or indirectly) the results of Phase III RCTs of biologics in CRSwNP and showcase the key between‐study differences that can influence trial outcomes and affect between‐study comparisons. The existing indirect treatment comparisons and meta‐analyses comparing biologics for CRSwNP are not discussed in detail, as this viewpoint seeks to present the key arguments as to why such comparative analyses should be interpreted with caution, given the significant heterogeneity in RCT methods and reporting in CRSwNP.

## 2 Phase III Biologic Trials in CRSwNP—How Do They Differ?

### 2.1 Patient Populations

The inclusion criteria varied across the Phase III RCTs in CRSwNP [22, 23, 24, 25, 26, 27, 28, 29, 30, 31], particularly those related to disease severity and T2 inflammation (Table 1). The POLYP‐1/‐2 (omalizumab), OSTRO, ORCHID (both benralizumab), and WAYPOINT (tezepelumab) trials included a minimum Sino‐Nasal Outcome Test 22 (SNOT‐22) score for eligibility, thereby enriching the study populations with individuals with a higher disease burden. The ANCHOR‐1/‐2 (depemokimab) trials included a broad patient population, with symptom entry criteria of moderate‐to‐severe nasal obstruction (score ≥ 2 on a verbal response scale) and presence of ≥ 2 different CRSwNP symptoms, but with no minimum SNOT‐22 threshold. There were also differences between studies in prior NP‐removal surgery, prior systemic corticosteroid (SCS) use, intranasal corticosteroids (INCS) use, and markers of T2 inflammation (Table 1), again indicating variations in disease severity. SYNAPSE (mepolizumab) was the only study that required patients to have received prior NP‐removal surgery and be eligible for repeat surgery for entry, and MERIT (mepolizumab) was the only study that did not require patients to be on INCS or standardize all patients to INCS during a run‐in period (with the exception of Japanese patients in ANCHOR‐1/‐2). Differences in the INCS run‐in duration were also observed, ranging from 4 weeks (SINUS‐24/‐52 [dupilumab], POLYP‐1/‐2, SYNAPSE, ORCHID, and CROWNS‐2 [stapokibart]) to 8 weeks (ANCHOR‐1/‐2), and most studies standardized INCS specifically to mometasone furoate during run‐in.

**TABLE 1:** Phase III RCTs assessing efficacy and safety of biologics for CRSwNP.

	SINUS‐24/‐52 [25]	SYNAPSE [27]	POLYP‐1/‐2 [23]	OSTRO [28]	ORCHID^b^ [29, 30]	MERIT [31]	WAYPOINT [22]	ANCHOR‐1/‐2 [26]	CROWNS‐2 [24]
**Biologic**	Dupilumab	Mepolizumab	Omalizumab	Benralizumab	Benralizumab	Mepolizumab	Tezepelumab	Depemokimab	Stapokibart
**Study dates**	2016–2018	2017–2018	2017–2019	2018–2020	2019–2025	2021–2023	2021–2024	2022–2023	2022–2023
**Population**	Bilateral CRSwNP with symptoms despite INCS	Recurrent, refractory, severe bilateral CRSwNP symptoms, eligible for repeat surgery	Inadequately controlled bilateral CRSwNP despite INCS	Bilateral CRSwNP symptoms despite INCS	Bilateral CRSwNP	Bilateral CRSwNP/ECRS	Bilateral CRSwNP requiring surgery	Inadequately controlled bilateral CRSwNP	Severe uncontrolled bilateral CRSwNP
**Blood eosinophil count inclusion criteria**	No	No	No	No	> 2% or ≥ 150 cells/µL	> 2%, JESREC score ≥ 11	No, except for Chinese patients: 80% with JESREC score ≥ 11 (i.e., > 2%)	No	Prespecified enrollment target of ≥ 60% to have eosinophilic CRSwNP (defined as blood eosinophils of ≥ 6.9% (without asthma) or ≥ 3.7% (with asthma), eosinophil count ≥ 55 per high power field or a percentage of eosinophils ≥ 27% in polyp tissue biopsy samples from screening/run‐in
**Asthma inclusion criteria**	Prespecified enrollment goal of 50% with asthma, N‐ERD, or both	No	No	No	Yes (100%)	No	Prespecified goal of 50%–70% with asthma or N‐ERD	No	No
**Stratified randomization of participants**	By country; previous surgery; comorbid asthma or N‐ERD	By country	By region; comorbid asthma or N‐ERD	By region; comorbid asthma	Not published yet	By country; background INCS use	By region; previous surgery; comorbid asthma/N‐ERD	By country; previous surgery	By comorbid asthma; eosinophilic status; previous surgery
**CRSwNP symptoms and QoL (SNOT‐22) inclusion criteria**	Moderate–severe NC or NO (score ≥ 2 of 3) + either LoS or discharge	Severe symptoms: NO (VAS score > 5 of 10) and overall VAS symptom score (> 7 of 10) + either discharge, facial pain/pressure, or LoS	Moderate–severe NCS (score ≥ 2 of 3) + postnasal drip, discharge or LoS + SNOT‐22 ≥ 20	Ongoing NP symptoms, + moderate–severe NBS (score ≥ 2 of 3) + SNOT‐22 ≥ 30	Ongoing NP symptoms, moderate–severe NBS (score ≥ 2 of 3) + SNOT‐22 ≥ 20	NO VAS score > 5; either NB/ NC/NO, or discharge plus either facial pain/pressure or LoS	Ongoing CRSwNP symptoms, moderate–severe NCS (score ≥ 2 of 3) + SNOT‐22 ≥ 30	Moderate–severe NO (score ≥ 2 of 3); + either LoS or discharge + either NO/NB/NC or discharge + facial pain/pressure or LoS	Moderate–severe NC (score ≥ 2 or 3); NPS ≥ 5 (≥ 2 in each nasal cavity)
**Prior surgery or SCS use inclusion criteria**	Either	≥ 1 previous surgery	Neither	Either	Either	Either	Either	Either	Either
**INCS**	4‐week run‐in (mometasone furoate nasal spray, 100 µg in each nostril twice daily)	4‐week run‐in (mometasone furoate nasal spray, 100 µg in each nostril twice daily)	4‐week run‐in (mometasone furoate nasal spray, 200 µg twice daily)	5‐week run‐in (mometasone furoate nasal spray, 400 µg daily)	4‐week run‐in	Not required	5‐week run‐in (mometasone furoate 400 µg daily)	8‐week run‐in (incl. intranasal liquid steroid washing/douching; excl. Japan)	4‐week run‐in (≥ 80% adherence to mometasone furoate nasal spray, 100 µg in each nostril daily)
**Geographic footprint**	27 countries from Asia (Japan), Australia, Europe, North and South America	11 countries from Asia (South Korea), Australia, Europe, North and South America	15 countries from Europe and North America	8 countries from Europe and North America	17 countries from Asia, Australia, Europe, North and South America	3 countries in Asia and Europe (Japan, China, Russia)	10 countries from Asia (China, Japan), Europe, North America	16 countries from Asia (China, Japan), Europe, North and South America	China only
**Symptom‐based co‐primary endpoint and timing of assessments**	NCS (4‐point Likert scale), daily average over 4‐week periods	NO VAS (score 0–10), daily average over 4‐week periods	NCS (4‐point Likert scale), daily average over 4‐week periods	NBS (4‐point Likert scale), daily average over 2‐week periods	NBS (4‐point Likert scale), timing of assessment not disclosed	NO VAS (score 0–10), daily average over 4‐week periods	NCS (4‐point Likert scale), daily average over 2‐week periods	NO VRS (4‐point Likert scale), daily average over 4‐week periods	NCS (4‐point Likert scale), daily average over 4‐week periods
**Dosing interval**	2–4 weeks	4 weeks	2–4 weeks	4 (first 3 doses)–8 weeks	4 (first 3 doses)–8 weeks	4 weeks	4 weeks	26 weeks	2 weeks
**Duration**	24–52 weeks	52 weeks	24 weeks	56 weeks	56 weeks	52 weeks	52 weeks	52 weeks	24 weeks + 28‐week open‐label extension
**CRSwNP biologics available during trial^a^**	None	None	None	Dupilumab	Dupilumab Mepolizumab Omalizumab	Dupilumab Mepolizumab Omalizumab	Dupilumab Mepolizumab Omalizumab	Dupilumab Mepolizumab Omalizumab	Dupilumab Mepolizumab Omalizumab
**Conducted during COVID‐19**	No	No	No	No	Yes	Yes	Yes	Yes	Yes

Abbreviations: CRSwNP, chronic rhinosinusitis with nasal polyps; ECRS, eosinophilic chronic rhinosinusitis; EMA, European Medicines Agency; FDA, US Food and Drug Administration; INCS, intranasal corticosteroid; JESREC, Japanese Epidemiological Survey of Refractory Eosinophilic Chronic Rhinosinusitis; LoS, loss of smell; NB, nasal blockage; NBS, nasal blockage score; NC, nasal congestion; NCS, nasal congestion score; N‐ERD, non‐steroidal anti‐inflammatory exacerbated respiratory disease; NO, nasal obstruction; NP, nasal polyps; NPS, nasal polyp score; QoL, quality of life; RCT, randomized controlled trial; SCS, systemic corticosteroid; SNOT‐22, Sino‐Nasal Outcome Test‐22; VAS, visual analog scale; VRS, verbal response scale.

^a^ Based on FDA and/or EMA approvals.

^b^ The ORCHID trial of benralizumab versus placebo was terminated as it did not meet its co‐primary endpoints of change from baseline in total endoscopic NPS and mean NBS at Week 56 [29, 30].

Most studies had no eligibility restrictions relating to T2 biomarkers such as blood eosinophil count (BEC) or T2 comorbidities such as asthma or nonsteroidal anti‐inflammatory exacerbated respiratory disease (N‐ERD) (Table 1). The MERIT and ORCHID studies required patients to have raised BEC, while CROWNS‐2 included a prespecified enrollment target for ≥ 60% to have eosinophilic CRSwNP and WAYPOINT was designed so that 80% of the Chinese patients included had an eosinophilic endotype. ORCHID was the only study that required all patients to have comorbid asthma on entry, though SINUS‐24/‐52 included a prespecified enrollment target for 50% of patients to have asthma/N‐ERD whereas this target was 50%–70% in WAYPOINT.

**FIGURE 1:**
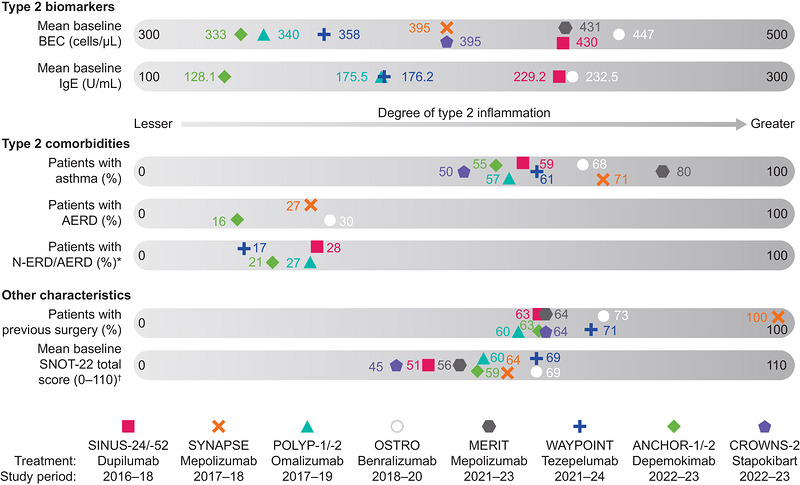
Baseline characteristics of patients from Phase III RCTs investigating biologics for the treatment of CRSwNP. Geometric means for baseline BEC are reported for the ANCHOR, SYNAPSE, and MERIT trials; arithmetic means for baseline BEC are reported for SINUS, POLYP, OSTRO, and WAYPOINT trials. IgE values were not reported for SYNAPSE, MERIT, or CROWNS‐2; N‐ERD data were not reported for MERIT or CROWNS‐2. Reported values for OSTRO, SYNAPSE (excluding Type 2 comorbidity data), POLYP‐1/2, MERIT, and CROWNS‐2 are approximate averages of each treatment group; values among the overall population were unavailable in published sources. Limited data from CROWNS‐2 have been included as the study was conducted in only one country (China). Details for ORCHID are not included in this figure as clinical characteristics data are currently unavailable. *The terminology used varied between studies (refer to original publications for further details); value shown for ANCHOR‐1/‐2 is the proportion of patients reported as having N‐ERD and/or AERD (unpublished data); ^†^total scores on SNOT‐22 range from 0 to 110, with higher scores indicating greater severity. AERD, aspirin‐exacerbated respiratory disease; BEC, blood eosinophil count; CRSwNP, chronic rhinosinusitis with nasal polyps; IgE, immunoglobulin E; N‐ERD, non‐steroidal anti‐inflammatory drug‐exacerbated respiratory disease; RCT, randomized controlled trial; SNOT‐22, Sino‐Nasal Outcome Test‐22.

Further, some trials stratified the randomization of patients by T2 comorbidities while others did not, risking an imbalance of these patients between placebo and active arms (Table 1). Reflecting these differences, baseline levels of indicators of T2 inflammation also differed across RCTs (Figure 1). The ANCHOR‐1/‐2 population, and to a lesser degree the POLYP‐1/‐2 population, had consistently lower rates of indicators of T2 inflammation (as measured using baseline BEC, IgE, and T2 comorbidities) than other RCT populations, while OSTRO and SINUS‐24/‐52 populations showed consistently higher rates.

These factors can significantly impact study outcomes and generalizability of the data. For example, patients with higher levels of T2 inflammation are suggested to be more responsive to biologic treatment [32, 33], and patients with more severe disease (versus those with milder disease) may (i) avoid potential ceiling effects by having more room to improve or (ii) have more edematous polyps, meaning potential reductions in volume can be greater and result in an apparently greater response.

### 2.2 Trial Designs and Endpoints

Trial durations were similar at 52–56 weeks, in line with current US Food and Drug Administration (FDA) guidance [34] (Table 1). Notably, the POLYP‐1/‐2 and CROWNS‐2 studies included a 24/28‐week open‐label extension period after the initial 24‐week double‐blind period [23, 24]. Also, in line with current FDA guidance [34], all CRSwNP RCTs assessed nasal polyp score (NPS) as a co‐primary endpoint (at 24 weeks in POLYP‐1/‐2, SINUS‐24/‐52, and CROWNS‐2; at 52–56 weeks in other studies). While there was consistent use of a 0–4 NPS for each nostril, there were variations in how Grade 2/3 polyps were scored between trials, for example, Grade 3 criteria is listed as large polyps reaching the lower border of the inferior turbinate, or polyps medial to the middle turbinate (POLYP‐1/‐2, WAYPOINT, ANCHOR‐1/‐2), and large polyps reaching the lower border of the inferior turbinate or large polyps of Score 2 with additional large polyps medial to the middle turbinate (OSTRO). These variations in NPS grading could influence the reported magnitude of change in response to treatment, and hinder the ability to effectively compare NPS reductions between trials, as reductions may not be equivalent [35]. There were also differences in how the other co‐primary endpoint (nasal congestion, obstruction, or blockage) was assessed: SYNAPSE and MERIT used a 10‐point visual analog scale, whereas all other studies used a 4‐point Likert scale (verbal response) (Table 1). Additionally, the time over which this co‐primary endpoint was assessed varied between studies.

### 2.3 Geography and Timing

Most studies were global multicenter studies covering the Asia‐Pacific, European, and American (North or South) regions, while some were region‐specific such as MERIT (limited to Japan, China, and Russia), POLYP‐1/‐2 and OSTRO (European and American regions only), and CROWNS‐2 (China only) [22, 23, 25, 26, 27, 28, 29, 31]. Patients from Asia (particularly China) were underrepresented in earlier trials preceding MERIT, ORCHID, WAYPOINT, ANCHOR‐1/‐2 and CROWNS‐2. Recruitment settings also varied across academic hospitals, specialist clinics, and dedicated trial sites, as observed in ANCHOR‐1/‐2 [26]. These differences may influence standard of care and treatment availability, and other contextual factors such as nutrition, lifestyle, and socioeconomic status. Importantly, inflammatory endotype distribution differs geographically, with China reporting a higher prevalence of non‐T2 or mixed eosinophilic–neutrophilic CRSwNP patterns compared with Western cohorts [36, 37]. While some studies accounted for T2 inflammatory burden defined by BEC/Japanese Epidemiological Survey of Refractory Eosinophilic Chronic Rhinosinusitis score (ORCHID, MERIT, CROWNS‐2, and WAYPOINT), the majority did not (Table 1). Overall, such heterogeneity underscores the need for global multiregional studies that capture diverse healthcare systems, treatment landscapes, and endotype profiles, thereby providing data more representative of a broad, real‐world patient population.

Regarding the timing of studies, the ORCHID, MERIT, WAYPOINT, ANCHOR‐1/‐2, and CROWNS‐2 trials were conducted within a more evolved treatment landscape than earlier studies, as several biologics for CRSwNP had become commercially available and treatment guidelines had been updated accordingly [33]. This may have led to (i) some degree of selection bias, for example, recruiting patients with milder disease as those with severe disease may not be willing to enter a placebo‐controlled clinical trial while effective treatment options are already available (something which would also hamper recruitment in general) and (ii) later studies may have included patients who had previously not responded sufficiently to biologics. Several trials were also conducted during the COVID‐19 pandemic, when widespread measures were introduced to prevent viral illness. In particular, social distancing, lockdowns, and behavioral changes due to fear of COVID‐19 likely affected hospital access and reported incidence of surgery, as well as patient compliance to background medication. Additionally, pandemic measures dramatically reduced circulation of other common respiratory pathogens, lowering exposure to inflammatory triggers and thereby likely decreasing the risk of exacerbations for patients with airway diseases [38]. This was followed by a period of increased respiratory infections when restrictions were lifted, which correlated with an increase in exacerbations for patients with asthma [39]. Lastly, COVID‐19 is a respiratory infection that, similar to CRSwNP, can result in a loss of smell [40]. Coinfection could therefore result in an apparent loss of efficacy. Together, these factors likely hamper efficacy comparisons across studies conducted before, during, and after the pandemic.

### 2.4 Data‐Handling Strategies

Strategies for handling intercurrent events (an event that occurs after treatment initiation that affects either the interpretation or the existence of measurements associated with the outcome of interest, e.g., a patient requiring a course of SCS or surgery) in RCTs can be complex. Different approaches include treatment policy, hypothetical, composite, principal stratum, and “while on treatment” strategies [41, 42]. Each strategy can lead to different estimands, and while the choice of strategy should align with the clinical question of interest, only recently has the FDA provided guidance on data handling for CRSwNP [34]. Most commonly‐used strategies are treatment policy (outcome irrespective of intercurrent event), composite strategy (intercurrent event becomes part of the outcome, e.g., worst observation carried forward or worst possible score), and on‐treatment strategy (outcome up to time of the intercurrent event) [41].

Key differences in data handling strategies across the Phase III RCTs were (i) the tendency to use more conservative (worst possible) imputations for surgery intercurrent events in later studies (MERIT, OSTRO, WAYPOINT, ANCHOR‐1/‐2); (ii) the use of a treatment policy strategy for short courses of SCS for SYNAPSE, MERIT, and ANCHOR‐1/‐2, but a composite strategy for other studies (where reported); and (iii) the use of a composite strategy for ANCHOR‐1/‐2, WAYPOINT, and CROWNS‐2 for the use of disease‐modulating medications, which were not defined as intercurrent events in the other studies (Table 2). Most studies used a treatment policy strategy for treatment discontinuations. Though some trials stopped endpoint assessments when SCS was administered and imputed worst observation carried forward, others adhered to current FDA guidelines and allowed short SCS courses as standard of care (treatment policy), for example, in the MERIT, SYNAPSE, and ANCHOR‐1/‐2 trials [34]. This permission of SCS could increase responses in arms requiring more SCS (i.e., placebo arms; this was observed in SYNAPSE and ANCHOR‐1/‐2 [26, 27]), potentially diluting the treatment effect relative to other studies where a composite strategy was employed. Moreover, the use of different estimand strategies leads to different clinical questions for each study, hence any cross‐study comparisons should be made with caution.

**TABLE 2:** Data handling across the Phase III RCTs.

		Handling of intercurrent events for primary analysis			
Biologic	Trial name(s)	Short courses of SCS	Surgery	Disease‐modulating treatment^a^	Treatment discontinuation
FDA guidance [34]		Treatment policy	Composite (e.g., WOCF, worst possible)^b^	Composite (e.g., WOCF, worst possible)	Treatment policy
Dupilumab	SINUS‐24/‐52 [25]	WOCF	WOCF	N/A	Treatment policy
Mepolizumab	SYNAPSE [27]	Treatment policy	WOCF	N/A	Treatment policy
	MERIT [31]	Treatment policy	Worst possible	N/A	Treatment policy/hypothetical strategy^c^
Omalizumab	POLYP‐1/‐2 [23]	WOCF	WOCF	N/A	WOCF^d^
Benralizumab	OSTRO [28]	WOCF	Worst possible	Not reported	Treatment policy
	ORCHID [29]	Information not available^e^			
Tezepelumab	WAYPOINT [22]	WOCF	Worst possible	WOCF	Treatment policy
Depemokimab	ANCHOR‐1/‐2 [26]	Treatment policy	Worst possible	Worst possible	Treatment policy
Stapokibart	CROWNS‐2 [24]	WOCF	WOCF	WOCF	WOCF^f^

Abbreviations: AE, adverse event; FDA, US Food and Drug Administration; INCS, intranasal corticosteroid; N/A, not applicable; RCT, randomized controlled trial; SCS, systemic corticosteroid; WOCF, worst observation carried forward.

^a^ Disease‐modulating treatment is a term used by GSK only to refer to medication that may reduce blood eosinophils or Type 2 inflammation (some biologics, continuous SCS, and INCS). For WAYPOINT, this is based on patients receiving another biologic for NP and for CROWNS‐2; it applies to those receiving prohibited medications that would classify as disease‐modulating treatment. N/A refers to trials where other biologics for CRSwNP were not available during the study.

^b^ Composite strategies incorporate the event into endpoint and classify it as treatment failure [41].

^c^ Premature discontinuation of study treatment unrelated to the COVID‐19 pandemic was handled using a treatment policy strategy, while premature discontinuation of study treatment, change in background medication, or start of prohibited medication related to the COVID‐19 pandemic was handled using a hypothetical strategy.

^d^ If treatment discontinued due to progressive disease, AEs, or lack of efficacy.

^e^ ORCHID trial was terminated as it did not meet its co‐primary endpoints and therefore this information is not available.

^f^ If treatment discontinued due to lack of efficacy, the presence of a medical condition deemed by the investigator to present significant risk and/or prevent protocol compliance, any intolerable AE or laboratory abnormality, use of specified prohibited drugs, pregnancy, or failure to comply with protocol requirements or study procedures.

## 3 Summary and Conclusion

As demonstrated in this article, significant heterogeneity exists between the methods and reporting of RCTs in CRSwNP. As with all research, an inherent risk of bias exists; it is acknowledged that this viewpoint article is subject to similar limitations and we encourage readers to critically assess the presented viewpoints. While it is tempting to directly compare RCT results for different biologics in CRSwNP, making accurate treatment comparisons between RCTs is challenging. Therefore, any direct/indirect comparisons should be made with extreme caution and any trial data used in the appropriate context. Efforts to match patients with similar characteristics at baseline, in particular disease severity and T2 inflammatory markers, may help improve the accuracy of between‐study comparisons. However, the only definitive way to establish the comparative efficacy of biologics in CRSwNP is via head‐to‐head trials. Although, the biologics currently approved or under investigation in CRSwNP have each demonstrated clinical benefits, real‐world evidence is essential to determine optimal treatment strategies for patients and provides an important evaluation of biologic effectiveness. In addition, further research such as head‐to‐head comparisons and identifying predictors of response should help to improve CRSwNP disease management in the future (Supporting Information).

## Author Contributions

All authors contributed to the conception and design of this review article, in addition to writing, editing, and providing final approval of the submitted version of the article.

## Funding

Editorial support for this article was funded by GSK. The sponsor (GSK) was involved in the conceptualization and review of this manuscript. All authors reviewed each draft and had final responsibility for the decision to submit for publication.

## Conflicts of Interest

Marjolein Cornet has received speaker fees and participated in advisory board meetings for Regeneron Pharmaceuticals Inc., Sanofi Genzyme, GSK, ALK, and Stallergenes Greer. Peter W. Hellings reports research grants and/or lecture fees by GSK, Sanofi/Regeneron, Viatris, Stallergenes, and Novartis. Martin Desrosiers has received clinical trial funding from AstraZeneca, GSK, Probionase Therapies, and Sanofi; has participated in advisory boards for Regeneron Pharmaceuticals, Inc., and Sanofi; and holds equity in Probionase Therapies. Martin Wagenmann has received personal fees from Allergopharma, ALK‐Abelló, AstraZeneca, Celltrion, CSL Behring, Genzyme, GSK, HAL Allergie, MSD, NeilMed, Sanofi, and Stallergenes, and is a member of the executive committee of the German Society of Allergology and Clinical Immunology (DGAKI). His institution has received payments from ALK‐Abelló, AstraZeneca, EU, GSK, Novartis, Regeneron, Sanofi, and Takeda. Lee Tombs is a consultant who has received payment from GSK. Richard Follows, Luz Adriana Jimenez, Dawn Edwards, and Peter Howarth are employed by GSK and hold financial equities in GSK. Laura Walrave was an employee of GSK and held financial equities in GSK at the time of submission. Joseph K. Han has received consultancy fees from Sanofi, Regeneron, AstraZeneca, and GSK.

## Supporting information




**Supporting File 1**: alr70205‐sup‐0001‐SuppMat.docx.

## Data Availability

Data sharing is not applicable to this article as no datasets were generated or analyzed during the current study.
