# Supplementary material for: Differences in Biologic Clinical Trials for Chronic Rhinosinusitis With Nasal Polyps—Are We Comparing Apples With Oranges?
Source: Int Forum Allergy Rhinol. 2026 Jul 3;16(8):769–77. doi: 10.1002/alr.70205 (PMC13432639; doi:10.1002/alr.70205)
Supplement: Supplementary file 1 — Supporting File 1: alr70205‐sup‐0001‐SuppMat.docx. [file ALR-16-769-s001.docx]

## **Supporting information**

**Differences in Biologic Clinical Trials for Chronic Rhinosinusitis With Nasal Polyps – Are We Comparing Apples With Oranges?**

Marjolein Cornet, Peter W Hellings, Martin Desrosiers, Martin Wagenmann, Richard Follows, Laura Walrave, Luz Adriana Jimenez, Lee Tombs, Dawn Edwards, Peter Howarth, Joseph K Han

## **Plain language summary**

Chronic rhinosinusitis with nasal polyps (CRSwNP) is a disease where soft non-cancerous growths called polyps develop inside the nose. Patients have symptoms such as a blocked or runny nose, loss of smell and feeling pain or pressure in their face. In most patients, the disease is caused by an overactive immune response. Several treatment options for CRSwNP that target specific parts of the immune system involved in the development of the polyps have been tested in clinical trials. These targeted therapies, known as biologics, can reduce the size of nasal polyps and improve symptoms. However, comparing results from different trials to judge which treatment is best is not straightforward. Trials vary in the patients they include (such as differences in symptom severity, treatment history, and other characteristics), their design, and how improvements are judged and analyzed. This article highlights some of the key differences between clinical trials for CRSwNP treatments and considers how these could have influenced results. Considering these differences is important when trying to match the right treatment options to the right patients at the right time.
